# Three-dimensional transesophageal echocardiographic evaluation of pulmonary vein anatomy prior to cryoablation: validation with cardiac CT scan

**DOI:** 10.1186/s12947-023-00305-9

**Published:** 2023-04-19

**Authors:** Laszlo Tibor Nagy, Csaba Jenei, Timea Bianka Papp, Reka Urbancsek, Rudolf Kolozsvari, Agnes Racz, Arnold Peter Raduly, Richard Veisz, Zoltan Csanadi

**Affiliations:** 1 Department of Cardiology, Faculty of Medicine, University of Debrecen, 22 Zsigmond Moricz Boulevard, Debrecen, 4032 Hungary; 2 Department of Radiology, Faculty of Medicine, University of Debrecen, Debrecen, Hungary

**Keywords:** Three-dimensional transesophageal echocardiography, Pulmonary vein imaging, Computed tomography, Cryoablation

## Abstract

**Background:**

Anatomical characteristics of the left atrium and the pulmonary veins (PVs) may be relevant to the success rate of cryoballoon (CB)-ablation for atrial fibrillation (AF). Cardiac computed tomography (CCT) is considered as the gold standard for preablation imaging. Recently, three-dimensional transesophageal echocardiography (3DTOE) has been proposed for preprocedural assessment of cardiac structures relevant to CB-ablation. The accuracy of 3DTOE has not been validated by other imaging modalities.

**Objective:**

We prospectively evaluated the feasibility and the accuracy of 3DTOE imaging for the assessment of left atrial and PV structures prior to pulmonary vein isolation (PVI). In addition, CCT was used to validate the measurements obtained with 3DTOE.

**Methods:**

PV anatomy of 67 patients (59.7% men, mean age 58.5 ± 10.5 years) was assessed using both 3DTOE and CCT scan prior to PVI with the Arctic Front CB. The following parameters were measured bilaterally: PV ostium area (OA), the major and minor axis diameters of the ostium (*a* > *b*) and the width of the carina between the superior and the inferior PVs. In addition, the width of the left lateral ridge (LLR) between the left atrial appendage and the left superior PV. Evaluation of inter-technique agreement was based on linear regression with Pearson correlation coefficient (PCC) and Bland–Altman analysis of biases and limits of agreement.

**Results:**

Moderate positive correlation (PCC 0.5–0.7) was demonstrated between the two imaging methods for the right superior PV’s OA and both axis diameters, the width of the LLR and left superior PV (LSPV) minor axis diameter (*b*) with limits of agreement ˂50% and no significant biases. Low positive or negligible correlation (PCC < 0.5) was found for both inferior PV parameters.

**Conclusions:**

Detailed assessment of the right superior PV parameters, LLR and LSPV *b* is feasible with 3DTOE prior to AF ablation. This 3DTOE measurements demonstrated a clinically acceptable inter-technique agreement with those obtained with CCT.

**Graphical Abstract:**

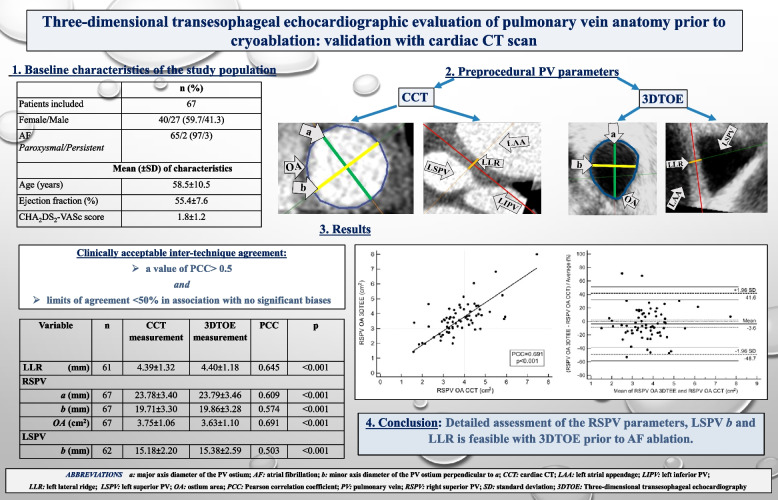

## Highlights

Novel 3DTOE method for the assessment of PV parameters relevant to CB ablation.

The first direct comparison of pre-ablation measurements with 3DTOE and CCT imaging. 3DTOE measurements of RSPV, LLR and the LSPV’s minor axis diameter were validated by CCT.

## Introduction

Pulmonary vein isolation (PVI) is a cornerstone of trancatheter ablation procedures for atrial fibrillation (AF). Although focal radiofrequency (RF) ablation guided by electroanatomical mapping has been considered as the „gold standard” technique for PVI, cryoballoon (CB) ablation has also gained popularity through recent years and has become the preferred technique in many centers. With either technology, precise information on left atrial (LA) and pulmonary vein (PV) anatomy before the ablation might be useful in planning the procedure including the selection of the most appropriate ablation technology. Because PV variants are common, the role of preablation imaging of PV and LA structures is controversial. This is the preferred approach in many centers arguing that a detailed knowledge of PVs and LA anatomy helps to determine the ablation strategy [1]. However, the results of other authors could not confirm that preablation CCT imaging would result in better outcome either with CB or RF ablation [2].

Cardiac computed tomography (CCT) or cardiac magnetic resonance (CMR) imaging are used rutinely for preablation evaluation of patients in many centers. The number of PVs, the size and geometry of the ostia, the width of the carina between the ipsilateral PVs and the width of the left lateral ridge (LLR) between the left atrial appandage (LAA) and the left sided PVs are considered significant predictors of successful isolation. Preprocedural assessment might provide especially useful information when PVI is planned with the CB, as the spherical balloon has potential limitations to accomodate to structures of different size and shape which may lead to incomplete occlusion, partial tissue contact and thereby isolation gaps along the PV perimeter [3–7]. Although these advanced three-dimensional (3D) imaging modalities are very useful for preablation evaluation, obvious drawbacks have to be considered related to their cost, the need for additional time and pre-arrangements and the radiation exposure in case of a CCT exam.

A potential alternative to preprocedural CCT imaging could be the use of intraprocedural intracardiac echocardiography (ICE). In this case the ablation technique could be selected based on the results of ICE used as the initial step of the invasive procedure. This approach could help to avoid the unfavorable outcome due to the anatomical features of the PVs. [8]. In order to further reduce the use of contrast material and fluoroscopic time, some authors recommend only ICE with color Doppler to confirm the first CB positioning [9].

PVs could also be visualised with transesophageal echocardiography ( TOE) in 90–100% of the cases in different prospective studies [10, 11]. Indeed, TOE has been used by several investigators [12–18] to guide the ablation procedure by providing real-time information on relavant cardiac structures and the relative position of the ablation device. Variable success rates have been reported and safety issues related to esophageal injury have also been raised by some of these studies [19–22] rendering the intraprocedural use of TOE a less preferred technique. However, TOE remains a potential option for preprocedural evaluation of PVs and related anatomy especially with recent progress in 3D reconstruction of TEE images ( 3DTOE). TOE is still routinely used by many centers to exlude the presence of an LAA thrombus prior to ablation, thereby it could be a reasonable substitute for CCT/CMR with no additional time and cost required.

Kettering et al. reported on the utilisation of 3DTOE in 30 patients [23], who, had undergone a first ablation with CB and were scheduled for a redo PVI. In another study by the same group, 3DTOE was used in 50 patiens [24] undergoing an initial PVI with RF or with CB technology. Excellent image quality was demonstrated in these investigations in the majority of the cases providing relevant anatomical information which facilitated the procedures with both ablation methods. Importantly, no direct comparison between the results obtained with 3DTOE versus with another 3D imaging modalities has been reported.

Herein, we prospectively evaluated the feasibility and the accuracy of 3DTOE for the assessment of the relevant LA and PV structures prior to PVI with the 2nd generation Arctic Front CB. In addition, CCT, considered the gold standard of cardiac imaging was also used in these patients in order to validate the measurements obtained with 3DTOE.

## Methods

### Study population

67 patients undergoing PVI with the 2nd generation CB for paroxysmal or persistent AF were included in the study. All patients had a CCT exam within 14 days and a 3DTOE exam within 24 h before the ablation procedure. All patients gave a written informed consent to the study, to both imaging methods and to CB ablation.

### CCT imaging

CCT was performed with GE Lightspeed VCT (GE Healthcare, USA, HU1008CT03) Single Source 64-detector computed tomography device. Retrospective electrocardiogram-triggered mode was applied at acquisition, tube current set at 300-750mAs (tissue density adjusted, peak current at 75% R-R interval), tube voltage at 100 kV. Omnipaque 350 mg/ml (GE Healthcare, USA) contrast solution at a dose of 50–60 ml with a rate of 5 ml/sec was administered, followed by 40 ml normal saline chaser at the same rate. Reconstruction and postprocessing were performed with 0.625 mm slice thickness using standard filter at the end of the T-wave, window settings at central 40/window 400, with slight adjustments if needed. Measurements were executed offline in multiplanar reconstruction mode with 3D viewer program (Volume Viewer™ 15.0 Ext.2). Data collection was performed when the best „en face” view was reached.

### 3DTOE examination and evaluation

#### Technical aspects

Our local protocol for visualization of the PV’s by 3DTOE has been reported [25]. Briefly, patients after a minimum 4-h fasting were examined in the left lateral decubitus position. Mild sedation was administrated using intravenous midazolam (2,5–5 mg). Philips EPIQ 7C echo scanner (Philips Healthcare, Andover, MA 01,810 USA) was used with X7-2t or X8-2t 3DTOE transducers. All studies were performed by two cardiologists (CsJ, LTN) with experience in echocardiography including 2D and 3DTOE.

First, LAA was visualized in 2D with upper (or mid) transoesophageal probe position at 20–45°, and the presence of an LA thrombus was excluded. The image was then centralized with a slight clockwise rotation to 60–80° to display the LAA together with the left superior PV (LSPV). 3D visualization was activated thereafter to allow recording of the LLR for offline measurements. In 2D mode, the probe angulation was changed to around 120° and then turned slightly counterclockwise in an anteflexion position from the LAA to visualize the branching of the left PVs. 3D mode was activated again to acquire the ostium of the LSPV and the left inferior PV (LIPV) as well as the left carina (LC) in between. To visualize the branching of the right PVs, the image was centered again at 45° in 2D mode, and the probe head was rotated clockwise maintaining the anteflexion position. After activation of the 3D mode, the right superior (RSPV) and the right inferior PV (RIPV) branches with the right carina (RC) were brought into sight and recorded. Slight changes in the patient’s left lateral decubitus position were allowed together with the rotation and the flexion of the probe during the examination if needed to improve the visibility of the PVs.

**Fig. 1:**
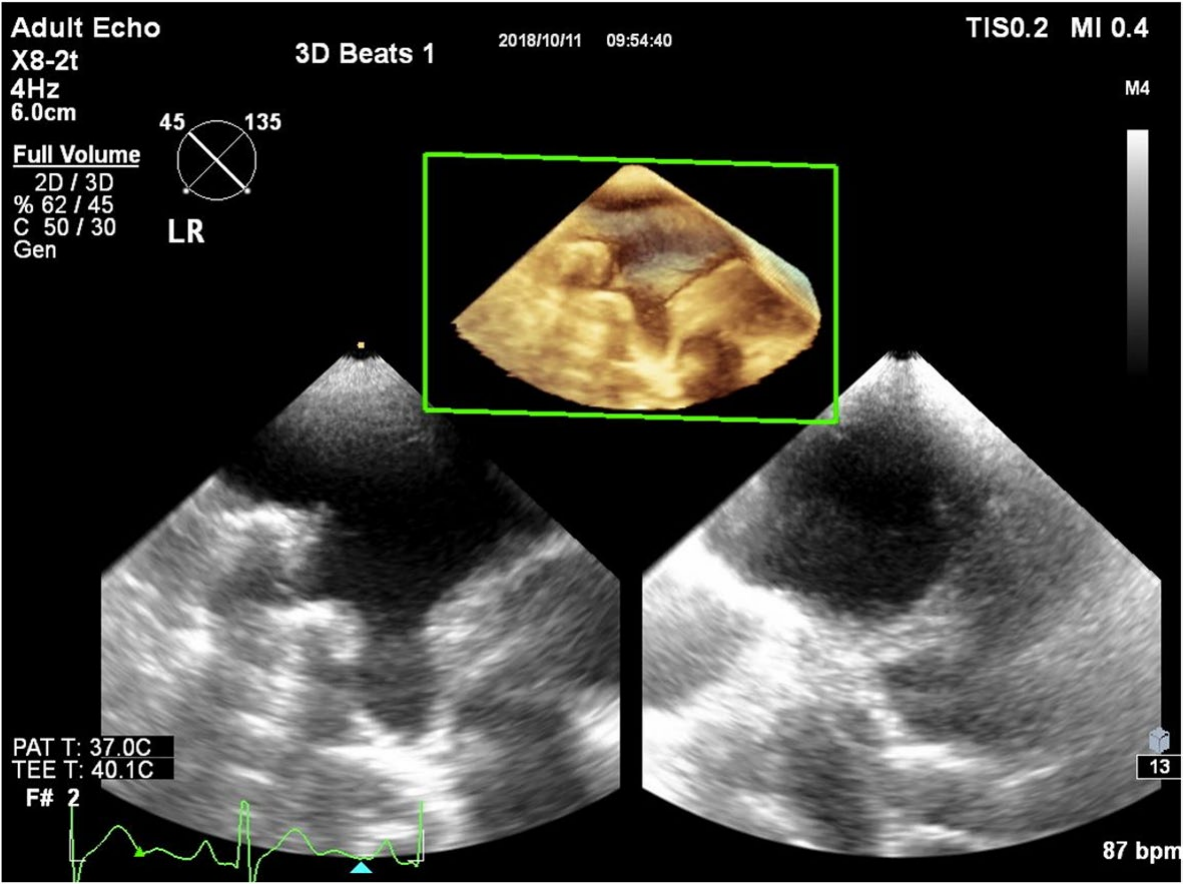
Acquisition of 3D dataset of right PVs. *3D* (Three-dimensional), *PV* (Pulmonary vein)

Effort was made to record multiple loops. Each loop contained two cardiac cycles. Figure 1 illustrates how the 3D dataset of PVs were acquired. The recorded 3D images were analyzed with QLab software (Philips Medical Systems) offline. The software was suitable for 3D multiplanar reconstruction (multiplanar review—MPR) capable of cutting full volume 3D datasets in any different plane. All measurements were done using an „en-face” view of the structure selecting a frame at the end of T wave. Recorded images were then evaluated and graded as to their suitability for further analysis as follows:

- *Grade I*: The whole cross-sectional "en face" view of the PV could be visualized on the reconstructed 3D image.
- *Grade II:* One third of the PV was missing on the reconstructed 3D cross-section image.
- *Grade III:* At least the half of the PV could not be displayed on the reconstructed 3D cross-section image.

Only grade I images were considered suitable for detailed PV measurements.

#### Measured parameters of LA structures

1. PV ostium parameters (Fig. 2):
  the major axis diameter of the PV ostium (*a*).
  the minor axis diameter of the PV ostium perpendicular to „*a*” (*b*).
  ostium area (OA) measured with planimetry.

**Fig. 2:**
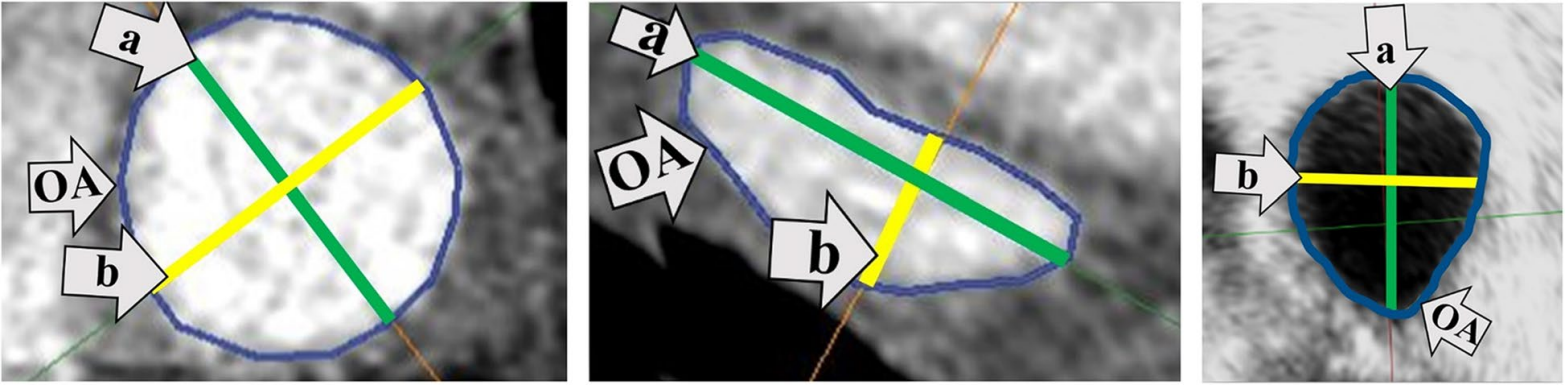
Measurement of PV ostium parameters. 2a left: Measurement of major (a, green line) and minor axis (b, yellow line) PV diameters as well as planimetric measurement of OA (blue line) from the „en face” 2D plane of a CCT image in case of a circular PV ostium. 2b middle: Measurement of the same parameters on a CCT image in case of an elliptic ostium. 2c right: Measurement of the same parameters on a 3DTOE image. PV (Pulmonary vein), a (The major axis diameter of the PV ostium), b (The minor axis diameter of the PV ostium perpendicular to „a”), *OA* (Ostium area), *2D* (Two-dimensional), *CCT* (Cardiac computed tomography), *3DTOE* (Three-dimensional transesophageal echocardiography)
2. Width of the LLR and the carina (Fig. 3):

**Fig. 3:**
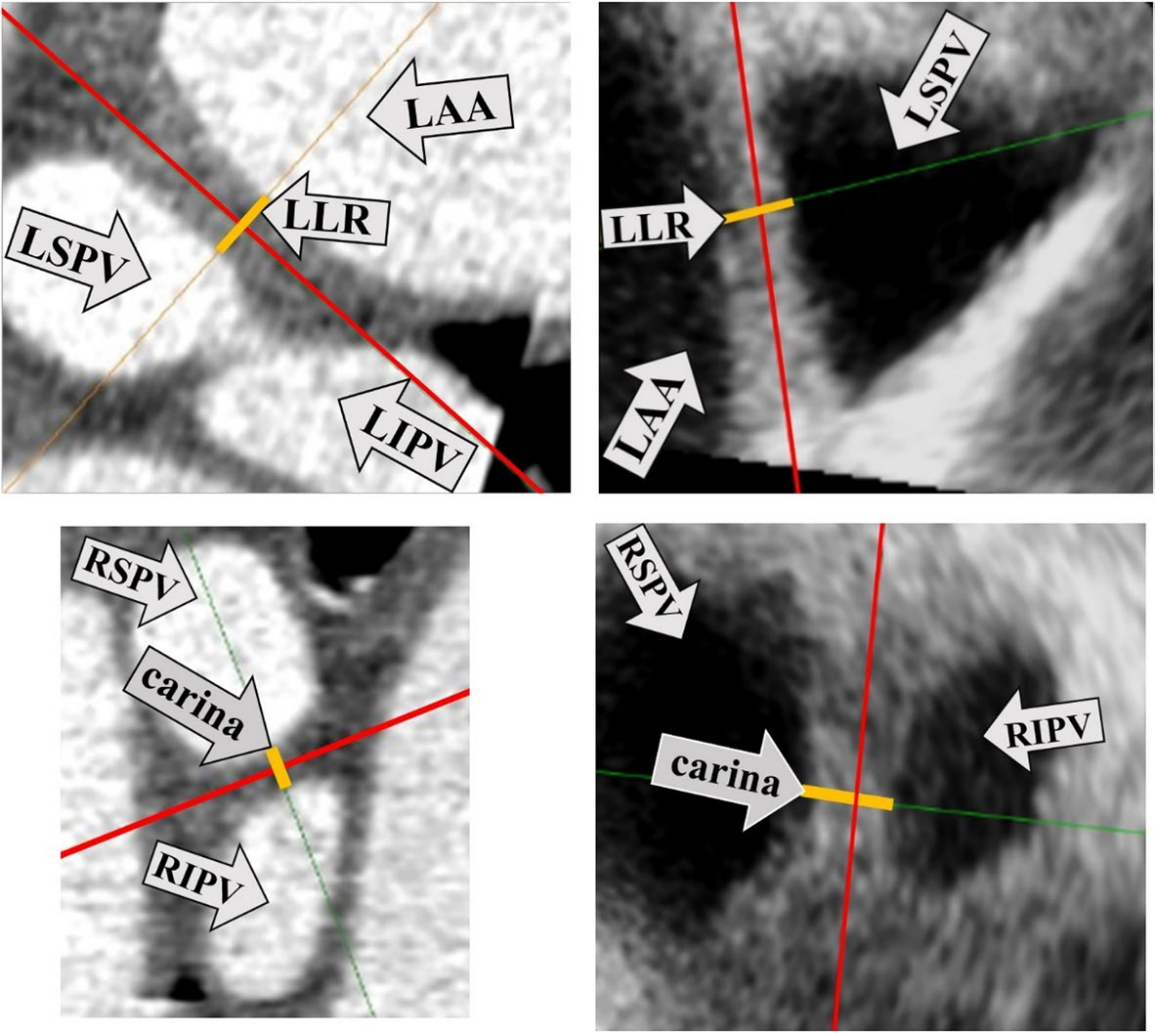
Measurement of the shortest distance (yellow line) perpendicular to the longitudinal axis (red line) of the LLR and the carina. Upper panel: Measurement of LLR width between the LSPV and the LAA from CCT (3a left) and from 3DTOE (3b right) images. Lower panel: Measurement of carina width between the RSPV and the RIPV from CCT (3c left) and from 3DTOE (3d right) images. *LLR* (Left lateral ridge), *LSPV* (Left superior PV), *LIPV* (Left inferior PV), *LAA* (Left atrial appendage), *CCT* (Cardiac computed tomography), *3DTOE* (Three-dimensional transesophageal echocardiography), *RSPV* (Right superior PV), *RIPV* (Right inferior PV)

#### Detection of anatomical PV variants

**Fig. 4:**
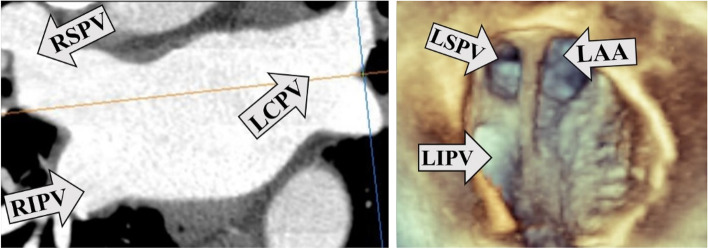
LCPV imaging. 4a left: LCPV detected by CCT in 2D longitudinal plane. 4b right: the same LCPV is not obvious on 3DTOE (full volume image, en face display). *LCPV* (Left common PV), *CCT* (Cardiac computed tomography), *2D* (Two-dimensional), *3DTOE* (Three-dimensional transesophageal echocardiography), *LSPV* (Left superior PV), *LIPV* (Left inferior PV), *LAA* (Left atrial appendage), *RSPV* (Right superior PV), *RIPV* (Right inferior PV)

**Fig. 5:**
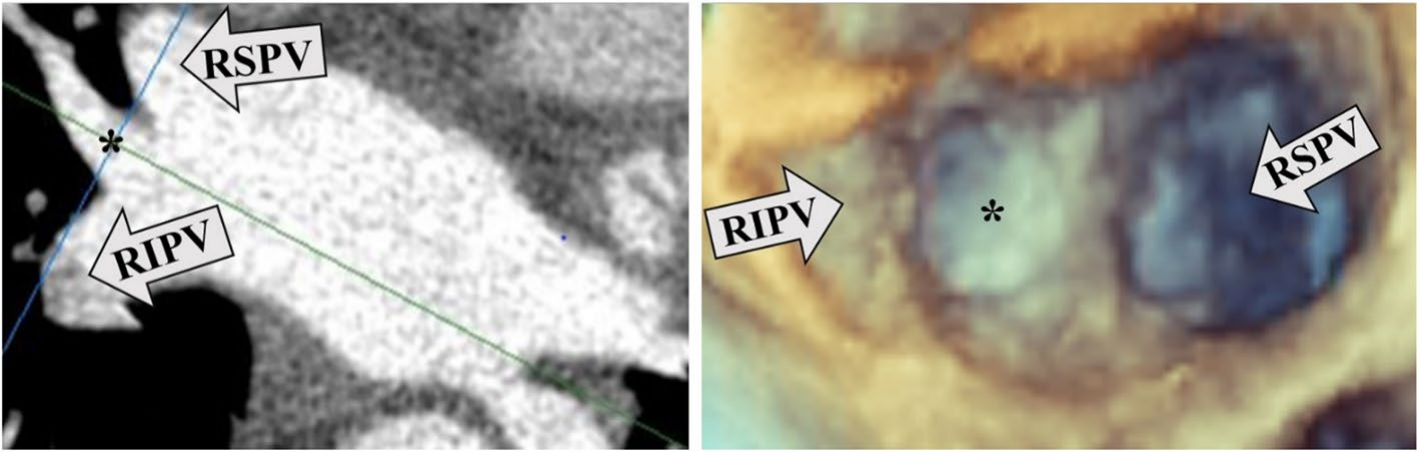
Supernumerary PV imaging. Supernumerary (right middle*) PV detected by CCT in 2D longitudinal plane (5a left) and by 3DTOE (full volume image, en face display; 5b right). *PV* (Pulmonary vein), *CCT* (Cardiac computed tomography), *2D* (Two-dimensional), *3DTOE* (Three-dimensional transesophageal echocardiography), *RSPV* (Right superior PV), *RIPV* (Right inferior PV)

Effort was made to detect the following anatomical variants with both imaging modalities: 1, common trunks including on the left (LCPV) and on the right side; 2, more than two (supernumerary) veins on either the left or on the right side (Figs. 4, 5).

#### Statistical analysis

For statistical analysis, we used IBM SPSS Statistics 26 program and MedCalc software version 13.3.3.0. All categorical variables were summarised as proportions and continuous variables as mean ± standard deviation (SD).

We selected randomly 17 patients in which 2 different operators (LTN, RU) performed separately and in blind manner a set of PV measurements both for 3DTOE and CCT. Interobserver reproducibility at 95% confidence interval (CI) was determined from the intraclass correlation coefficient (ICC) together with its corresponding *p*-value.

Inter-technique agreement between the two imaging methods was evaluated using linear regression with Pearson’s correlation. The following rule of thumb for interpreting the size of the Pearson correlation coefficient (PCC) was used: 0.90 to 1.00- very high positive correlation, 0.70 to 0.90—high positive correlation, 0.50 to 0.70—moderate positive correlation, 0.30 to 0.50—low positive correlation, 0.00 to 0.30- negligible correlation [26, 27].

PV anatomical parameters with a value of PCC > 0.5 were further tested to determine the biases and limits of agreement using Bland–Altman plot [28–30].

## Results

### Baseline patient characteristics

67 patients (age: 58.5 ± 10.5 years; 59.7% man) undergoing AF ablation were enrolled. Baseline clinical parameters of the patients are listed in Table 1***.*** The majority of patients (97%) had paroxysmal AF. The most common comorbidities included hypertension (53.7%), diabetes mellitus (16.4%) and peripheral vascular disease (10.4%). The mean CHA_2_DS_2_-VASc score was 1.8 ± 1.2.

**Table 1:** Baseline characteristics of the study population

	**n (%)**
Patients included	67
Female/Male	40/27 (59.7/41.3)
AF *Paroxysmal/Persistent*	65/2 (97/3)
	**Mean (± SD) of characteristics**
*Age (years)*	58.5 ± 10.5
*Ejection fraction (%)*	55.4 ± 7.6
*CHA*_*2*_*DS*_*2*_*-VASc score*	1.8 ± 1.2

*AF* Atrial fibrillation, *SD* Standard deviation, *CHA*_*2*_*DS*_*2*_*-Vasc score* C- Congestive heart failure, *H* Hypertension > 140/90 mmHg, *A* Age ≥ 75 years, *D* Diabetes mellitus, *S* Stroke, *V* Vascular disease, *A* Age 65–74 years, *Sc* Sex category

#### Grading of 3DTOE PV imaging

Fifty-six out of 67 patients (83.6%) were in sinus rhythm during 3DTOE exam. Image quality obtained for the different PVs according to our grading system are displayed in Table 2. The highest ratio of the optimal image quality was obtained for RSPV (100%), while the lowest (83.6%) for LIPV.

**Table 2:** Grading of 3DTOE PV image quality

	**LSPV**	**LIPV**	**RSPV**	**RIPV**
	**n (%)**			
*Grade I*	64 (95.5)	56 (83.6)	67 (100)	62 (92.5)
*Grade II*	1 (1.5)	4 (6.0)	0 (0)	2 (3.0)
*Grade III*	2 (3.0)	7 (10.4)	0 (0)	3 (4.5)

Grade I: The whole cross-sectional "en face" view of the PV could be visualized on the reconstructed 3D image, Grade II: One third of the PV was missing on the reconstructed 3D cross-section image, Grade III: At least the half of the PV could not be displayed on the reconstructed 3D cross-section image

*3D* Three-dimensional, *3DTOE* Three-dimensional transesophageal echocardiography, *PV* Pulmonary vein, *LSPV* Left superior PV, *LIPV* Left inferior PV, *RSPV* Right superior PV, *RIPV* Right inferior PV

#### Determination of interobserver agreement for 3DTOE and CCT PV measurements

For all PV measurements, the ICC ranges of the interobserver evaluations were 0.87–0.99 with *p* = 0.001. For 3DTOE measurements the highest ICC was calculated for RSPV OA (ICC = 0.99, 95% CI: 0.97–0.99, *p* = 0.001), while the lowest ICCs were obtained for LIPV OA (ICC = 0.88, 95% CI:0.69–0.96, *p* = 0.001) and for LC (ICC = 0.88, 95% CI:0.71–0.95, *p* = 0.001). For CCT measurements the highest ICCs were demonstrated for LSPV *b *(ICC = 0.98, 95% CI: 0.94–0.99, *p* = 0.001), for RSPV OA (ICC = 0.98, 95% CI: 0.95–0.99, *p* = 0.001) and for RSPV *b *(ICC = 0.98, 95% CI: 0.95–0.99, *p* = 0.001). The lowest ICC value was found for LIPV *a *(ICC = 0.87, 95% CI:0.68–0.95, *p* = 0.001).

#### Evaluation of the agreement between the two imaging techniques

Our definition of a clinically acceptable inter-technique agreement between the two imaging methods was a PCC > 0.5 together with limits of agreement < 50% and no significant biases.

### Pearson’s linear regression

Measurements related to the PV anatomy with PCC and *p* values are displayed in Table 3***.*** A high positive correlation (PCC 0.7–0.9) was demonstrated in the absence of anatomical variants for the width of both carinas. A moderate positive correlation (PCC 0.5–0.7) was found for OA, *a* and* b* values of both superior PVs and for the LLR width. However RIPV OA, RIPV *b* and all LIPV parameters showed a low positive (PCC 0.3–0.5), while RIPV *a* a negligible (PCC 0.0–0.3) correlation.

**Table 3:** Measurements related to the PV anatomy with PCC and associated *p* values

**Variable**		**n**	**CCT measurement**^a^	**3DTOE measurement**^a^	**PCC**^d^	***p***^e^
**LC**^b^	**(mm)**	48	5.71 ± 2.89	5.23 ± 2.48	0.878	< 0.001
**RC**^c^	**(mm)**	46	6.24 ± 2.09	6.64 ± 2.02	0.748	< 0.001
**LLR**	**(mm)**	61	4.39 ± 1.32	4.40 ± 1.18	0.645	< 0.001
**RSPV**						
**a (mm)**		67	23.78 ± 3.40	23.79 ± 3.46	0.609	< 0.001
**b (mm)**		67	19.71 ± 3.30	19.86 ± 3.28	0.574	< 0.001
**OA (cm**^**2**^**)**		67	3.75 ± 1.06	3.63 ± 1.10	0.691	< 0.001
**LSPV**						
**a (mm)**		64	21.12 ± 2.74	20.08 ± 2.79	0.556	< 0.001
**b (mm)**		62	15.18 ± 2.20	15.38 ± 2.59	0.503	< 0.001
**OA (cm**^**2**^**)**		64	2.50 ± 0.55	2.32 ± 0.65	0.545	< 0.001
**RIPV**						
**a (mm)**		62	20.10 ± 2.83	23.01 ± 3.36	0.290	0.022
**b (mm)**		62	16.84 ± 2.78	15.54 ± 2.72	0.403	0.001
**OA (cm**^**2**^**)**		62	2.71 ± 0.80	2.57 ± 0.67	0.466	< 0.001
**LIPV**						
**a (mm)**		54	18.39 ± 1.93	17.94 ± 3.02	0.367	0.006
**b (mm)**		54	13.36 ± 3.11	12.25 ± 2.56	0.364	0.007
**OA (cm**^**2**^**)**		54	1.89 ± 0.54	1.56 ± 0.51	0.497	< 0.001

*PV* Pulmonary vein, *CCT* Cardiac computed tomography, *3DTOE* Three-dimensional transesophageal echocardiography, *a* The major axis diameter of the PV ostium, *b* The minor axis diameter of the PV ostium perpendicular to „a”, *OA* Ostium area, *LC* Left carina, *RC* Right carina, *LLR* Left lateral ridge, *LSPV* Left superior PV, *LIPV* Left inferior PV, *RSPV* Right superior PV, *RIPV* Right inferior PV, *LCPV* Left common PV, *PCC* Pearson correlation coefficient, *SD* Standard deviation

^a^Values are presented as mean ± SD

^b^In the absence of LCPV and left supernumerary PV

^c^In the absence of right supernumerary PV

^d^In the case of PCC 0.50–0.90, the measurements were considered to have a moderate to high positive correlation

^e^*p* value of the PCC considered statistically significant if < 0.001

### Bland–Altman analysis

**Fig. 6:**
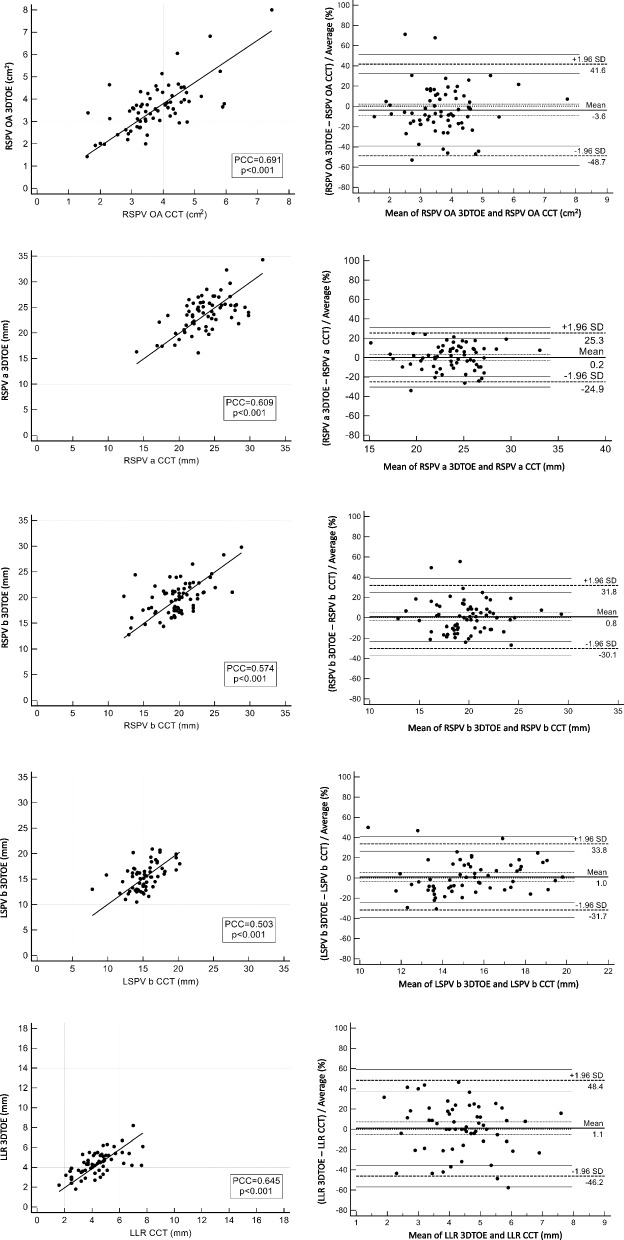
Pearson’s linear regressions of PV parameters with a value of PCC > 0.5 together Bland–Altman plots with limits of agreement < 50% and no significant biases. The consecutive graphs (6 a, b, c, d, e) represent the Pearson’s correlation and Bland–Altman analysis of the PV parameters for which our clinically acceptable moderate inter-technique agreement criteria were met. Bland–Altman plots of differences between 3DTOE and CCT, expressed as percentages of the values on the axis [(3DTOE – CCT) /average %)], vs. the mean of the two measurements. Areas between continuous lines present CI limits for agreement limits. Area between dashed lines presents CI limits for mean limits. The bias is not significant because the zero line is within the CI limits for mean limits. The 95% limits of agreement are presented as ± 1.96 SD. *3DTOE* (Three-dimensional transesophageal echocardiography), *CCT* (Cardiac computed tomography), *PV* (Pulmonary vein), a (The major axis diameter of the PV ostium), b (The minor axis diameter of the PV ostium perpendicular to „a”), *OA* (Ostium area), *LLR* (Left lateral ridge), *LSPV* (Left superior PV), *RSPV* (Right superior PV), *PCC* (Pearson correlation coefficient), *CI* (Confidence interval), *SD* (Standard deviation)

PV anatomical parameters with a value of PCC > 0.5 were further tested by Bland–Altman analysis. Bland–Altman plots demonstrated limits of agreement < 50% and no significant biases for the following PV parameters: RSPV OA, RSPV *a*, RSPV *b*, LSPV *b* and LLR. Figure 6 shows the Pearson’s correlation and Bland–Altman analysis of PV parameters fulfilling our inter-technique agreement criteria.

#### 3DTOE imaging of anatomical variants of the PVs

Anatomical variants of the PVs were detected with the CCT, which is considered the gold standard. With echocardiography, right supernumerary PVs were correctly identified in 37.5%, while LCPVs were identified in only 8.3% of the cases (Table 4).

**Table 4:** Visualization of PV anatomic variations with the two imaging techniques

	**3DTOE (n)**	**CCT (n)**	**assessed with 3DTOE (%)**
***Anatomic variations***			
*LCPV*	1	12	8.3
*Left supernumerary PV*	0	1	0
*Right supernumerary PV*	3	8	37.5

*PV* Pulmonary vein, *3DTOE* Three-dimensional transesophageal echocardiography, *CCT* Cardiac computed tomography, *LCPV* Left common PV

## Discussion

### Main findings

This is the first prospective direct comparison of preprocedural 3DTOE and CCT for visualisation and numerical characterisation of PV anatomy by measuring multiple parameters potentially relevant to the success of PVI with CB. We also tested the TOE acquisiton technique developed by our group specifically for the optimal 3DTOE image acquisition and visualisation of the PV structures. Based on the fact that clinically acceptable inter-technique agreement was demonstrated for all RSPV, LSPV b and LLR parameters, 3DTOE provides reliable data on anatomical features of superior PVs that may be important for CB ablation. However the accuracy of 3DTOE in the presence of anatomical variants and for the assessment of both inferior PVs was not acceptable.

### Methodological considerations for preprocedural PV imaging

The ROTEA study prospectively compared different PV measurements obtained with 2DTOE versus with CCT [31] before and after AF ablation. 2DTOE consistently underestimated the maximum ostial dimensions compared with CCT, especially for the inferior PVs, likely due to the limited field of view resulting from the close proximity of the TOE probe to these cardiac structures. Further, LCPVs revealed by CCT were identified with TOE in only 54% of the cases. This could also be explained by the near-field view limiting a clear definition of the PV-LA junction [32, 33]***.***

In our research, 3D reconstruction obtained in the left lateral decubitus position yielded excellent (grade I) image quality in 100%, 95.5%, 92.5% and 83.6% for RSPV, LSPV, RIPV and LIPV, respectively (Table 2). Anatomical variants on the right side could be visualised at a low, but still significantly higher rate (37.5%) than on the left (7.7%; Table 4). These observations are in line with the findings of Faletra et al. [12], who considered the distance between the esophagus and the ipsilateral PVs as a major limiting factor of proper visualisation.

In recent years, advances in electronic and transducer technology have allowed the development and successful implementation of real-time 3D ICE. This tool could overcome the limitations mentioned above by displaying PV structures more precisely even in the case of complex anatomy [34].

### Potential clinical applications

3DTOE PV assessment with the technique described in this study can be performed as part of the routine preablation TOE exam to exclude LAA thrombus. Incorporation of 3DTOE data in 3D electroanatomical mapping systems would further expand the use of this approach as a cheaper and simpler method for preablation evaluation of patients.

## Limitations

This was a single-center study with a relatively small number of patients.

## Conclusion

Detailed assessment of the RSPV parameters, LSPV *b* and LLR is feasible with 3DTOE prior to AF ablation. This 3DTOE measurements demonstrated a clinically acceptable moderate inter-technique agreement with those obtained with CCT. Prospective studies are required to assess the predictive value of preablation 3DTOE PV measurements validated by CCT for the clinical outcome of CB ablation.

## Data Availability

The datasets used and/or analysed during the current study are available from the corresponding author on request.

## Abbreviations

2D: Two-dimensional
3D: Three-dimensional
3DTOE: Three-dimensional transesophageal echocardiography
*a*: Major axis diameter of the PV ostium
AF: Atrial fibrillation
*b*: Minor axis diameter of the PV ostium perpendicular to*,,a”*
CB: Cryoballoon
CCT: Cardiac computed tomography
CI: Confidence interval
CMR: Cardiac magnetic resonance
ICC: Intraclass correlation coefficient
ICE: Intracardiac echocardiography
LA: Left atrial
LAA: Left atrial appendage
LC: Left carina
LCPV: Left common PV
LIPV: Left inferior PV
LLR: Left lateral ridge
LSPV: Left superior PV
MPR: Multiplanar review
OA: Ostium area
PCC: Pearson correlation coefficient
PV: Pulmonary vein
PVI: Pulmonary vein isolation
RC: Right carina
RF: Radiofrequency
RIPV: Right inferior PV
RSPV: Right superior PV
SD: Standard deviation
TOE: Transesophageal echocardiography
